# Reduced cortical volume of the default mode network in adolescents with generalized anxiety disorder

**DOI:** 10.1002/da.23252

**Published:** 2022-03-21

**Authors:** Johannah Bashford-Largo, Ru Zhang, Avantika Mathur, Jaimie Elowsky, Amanda Schwartz, Matthew Dobbertin, Robert James R. Blair, Karina S. Blair, Sahil Bajaj

**Affiliations:** 1 Multimodal Clinical Neuroimaging Laboratory (MCNL), Center for Neurobehavioral Research, Boys Town National Research Hospital, Boys Town, Nebraska, USA; 2 Center for Brain, Biology, and Behavior, University of Nebraska-Lincoln, Lincoln, Nebraska, USA; 3 Department of Psychology, University of North Dakota, Grand Forks, North Dakota, USA; 4 Inpatient Psychiatric Care Unit, Boys Town National Research Hospital, Boys Town, Nebraska, USA; 5 Child and Adolescent Mental Health Centre, Mental Health Services, Capital Region of Denmark, Copenhagen, Denmark

**Keywords:** adolescents, cortical volume, Default Mode Network, GAD, neuroimaging

## Abstract

**Background:**

Widespread structural alterations have been shown to be implicated in individuals with generalized anxiety disorder (GAD). However, there have been inconsistent findings in cortical volume (CV) differences. Most structural neuroimaging studies looking at GAD used region-based approach with relatively small sample sizes, let alone be specific to adolescents with GAD. We believe this is the first study to look at CV measures using a network-based approach in a larger sample of adolescents with GAD. The goal of the current study was to focus on three different brain networks (i.e., Limbic, Frontoparietal, and Default Mode Network [DMN]) in adolescents with GAD.

**Method:**

The study involved 81 adolescents with GAD and 112 typically developing (TD) comparison individuals matched on age (15.98 and 15.63 respective means), sex (42F/39M and 45F/67M), and IQ (101.90 and 103.94 respective means). Participants underwent structural MRI. Freesurfer was used to estimate CV (both network-specific and region-specific within networks) and region-specific subcortical volume measures. Multivariate analysis of covariance (MANCOVA; with sex, age, IQ, and intracranial volume [ICV] as potential covariates) was used to estimate group differences.

**Results:**

We found significantly lower CV for the DMN in adolescents with GAD, compared with TD individuals. Adolescents with GAD also showed significantly lower hemispheric mean CV of the default-mode regions (particularly the prefrontal and temporal regions) and the hippocampus, compared with TD individuals.

**Conclusion:**

The current findings suggest structural alterations in adolescents with GAD. These structural alterations will need to be addressed when implementing and developing treatments for patients with GAD.

## 1 | INTRODUCTION

Generalized anxiety disorder (GAD) is an anxiety disorder associated with excessive worry. Prognosis is poor as most adult anxiety or depressive disorders are preceded by anxiety disorders in adolescence. Moreover, the pathophysiology underpinning GAD remains relatively unclear. Functional neuroimaging studies have been conducted revealing dysfunction related to emotion processing (Blair et al., 2012; Etkin et al., 2010; Monk et al., 2008; Waters et al., 2008), working memory (Moon & Jeong, 2017), reward processing (Bashford-Largo et al., 2021; DeVido et al., 2009), and top-down attentional control (Bashford-Largo et al., 2020; Blair et al., 2012). These studies have particularly implicated the prefrontal cortex (PFC), hippocampus, and amygdala in the pathophysiology of GAD (Etkin & Wager, 2007). However, the extent to which these atypical functional findings are mirrored by brain structural alterations in patients with GAD remains unclear. The current study explores the pathophysiology underpinning GAD using network-based structural magnetic resonance imaging (sMRI).

Prior studies link GAD to structural alterations in regions including the PFC, amygdala, and hippocampus (Kolesar et al., 2019; Madonna et al., 2019). However, the findings are relatively inconsistent. No regions have been implicated as atypical in over 50% of the sMRI studies conducted though it should be noted that many of the studies involved group-specific regions of interest (ROI) (De, 2000; Etkin et al., 2009; Milham et al., 2005). With respect to PFC, GAD in adolescents has been related to increased anterior cingulate (Strawn et al., 2015) and ventral medial prefrontal cortex (vmPFC) (Gold et al., 2017) volumes and GAD in adults to increased dorsal medial prefrontal cortex (dmPFC) volumes (Schienle et al., 2011). However, there have also been reports linking GAD in adolescents to *decreased* volumes within orbitofrontal cortex (OFC) (Strawn et al., 2013), inferior frontal gyrus (IFG) (Strawn et al., 2015), and dorsolateral prefrontal cortex (dlPFC) (Hilbert et al., 2014) and GAD in adults to decreased volumes within middle frontal gyrus (MFG) (Molent et al., 2018) and dlPFC (Moon & Jeong, 2017). The lack of regional consistency in the above findings may partly reflect the relatively small sample sizes involved in those studies. Only one of those studies involved more than 50 patients with GAD (Gold et al., 2017). However, it is noteworthy that most of the regions reported to be implicated in GAD are either part of the limbic, frontoparietal, or DMNs (Bajaj et al., 2017).

In addition to the lack of consistency of regions identified by the sMRI studies, there is a marked lack of replication in the direction of the findings with reports of both increased and decreased cortical volumes in GAD. Some were noted above in relation to the PFC findings described above. Subcortical findings are largely similar. For example, increased amygdala volumes were reported in two studies involving adolescents with GAD (De, 2000; Schienle et al., 2011) and one study involving adults with GAD (Etkin & Wager, 2007). However, another two studies reported *reduced* amygdala volumes in adolescents with GAD (Milham et al., 2005; Strawn et al., 2013). There are relatively consistent findings for variation in subcortical volume of hippocampus in GAD patients; a majority of studies have reported reduction in hippocampal volume in adolescents or adults with GAD (Gold et al., 2017; Hettema et al., 2012; Moon et al., 2014). However, many other studies have not reported hippocampal structural abnormalities in patients with GAD (De Bellis et al., 2000; De Bellis et al., 2002; Milham et al., 2005).

The choice of dependent measures for sMRI studies is also important. The three most commonly used morphometric measures are cortical thickness (CT), cortical surface area (CSA), and CV. Large volume is accompanied by a smaller increase in thickness and relatively large increase in area (Pakkenberg & Gundersen, 1997; Winkler et al., 2010). While analyzing CT and CSA individually may improve the specificity compared with combined metric (i.e., CV), the joint analysis of CT and CSA in terms of CV may be potentially more informative as it increases the power to simultaneously quantify the effects of CT and CSA (Rimol et al., 2012). Therefore, in the current study, we used CV as our primary parameter of interest, whereas in follow-up analysis, we explored the group differences in CT and CSA as well. While it is important to identify region-specific structural changes associated with GAD, it is even more important to focus on structural changes in both networks as well as in their respective regions. This should improve the interpretability of findings relative to reports of structural abnormalities in specific brain regions individually.

In this study, our primary goal was to look at the differences in CV in adolescents with GAD in three different networks: the limbic network (LN), frontoparietal network (FN), and default-mode network (DMN), *relative* to typically developing (TD) adolescents. Given the previous literature and the fact that previous work has not taken a network-based approach to examining potential CV differences in patients with GAD, we made two predictions. With respect to the network-level data, we hypothesized that there would be reduced CV in the GAD group relative to the TD group, potentially particularly within the limbic, frontoparietal, and DMNs. Based on the relative inconsistency with respect to findings on the amygdala and consistency with respect to the hippocampus, we predicted that the hippocampus would show reduced volumes for the GAD group relative to the TD group.

## 2 | METHODS

### 2.1 | Participants

The current study included data collected from 193 youths between 10 and 19 years of age (mean age = 15.78 ± 1.71 years, 106 males). Participants were recruited from a residential care facility at Boys Town National Research Hospital (BTNRH) and from the surrounding community. Participants recruited from the residential facility had been referred for behavioral and mental health problems whereas participants from the community were recruited through flyers or social media. There were two groups of participants: adolescents with clinically significant levels of GAD (GAD group; *N* = 81; 39 males; mean age = 15.98 ± 1.65 years; mean GAD score = 9.68 ± 4.95) and TD adolescents as controls (TD group; *N* = 112; 67 males; mean age = 15.63 ± 1.74 years; mean GAD score = 4.03 ± 3.01). Exclusion criteria included braces, claustrophobia, active substance dependence, pervasive developmental disorder, Tourette’s syndrome, lifetime history of psychosis, neurological disorder, head trauma, non-English speaking, and presence of active safety concerns. Clinical characterization was done through psychiatric interviews by licensed and board-certified child and adolescent psychiatrists with the participants and their parents to adhere closely to common clinical practice. All participants and their parents provided written informed assent/consent before enrollment. The study protocol was approved by the Institutional Review Board at BTNRH.

### 2.2 | Data collection

#### 2.2.1 | Neuroanatomical data

High-resolution sMRI (T1-weighted) data were collected using 3 T Siemens MRI scanner located at BTNRH. Each participant was instructed to rest, relax, and try their best to minimize head movement during the entire scan. Whole-brain anatomical data for each participant were acquired using a 3D magnetization-prepared rapid acquisition gradient echo (MPRAGE) sequence, which consisted of 176 axial slices (slice thickness = 1 mm, voxel resolution = 0.9 × 0.9 × 1 mm^3^, repetition time = 2200 ms; echo time = 2.48 ms; matrix size = 256 × 208; field of view (FOV) = 230 mm, and flip angle = 8°).

#### 2.2.2 | General intelligence (IQ)

The Wechsler Abbreviated Scale of Intelligence II (WASI-II) (Wechsler, 2011) was used to estimate IQ in the domains of perceptual reasoning, verbal comprehension, and Full-Scale IQ (FSIQ). FSIQ scores have high reliability (*α* = .98) and strong correlations (*r* = .92) with scores on the full Wechsler Adult Intelligence Scale (WAIS)-III (Wechsler, 1997, 1999) and were used in the current context.

#### 2.2.3 | Screen for Child Anxiety Related Emotional Disorder (SCARED) scale

SCARED (child version; Birmaher et al., 1997) is a self-report questionnaire that looks at a youth’s potential for having an anxiety condition (GAD, panic disorder, separation anxiety disorder, social anxiety disorder, and school anxiety). Prior work has indicated that the SCARED has excellent internal consistency and test–retest reliabilities (*α* = .921 and *r* = .782 for random effects model) (Runyon et al., 2018).

#### 2.2.4 | The Mood and Feelings Questionnaire (MFQ)

MFQ (Costello & Angold, 1988) is a self-report questionnaire that assesses various symptoms of depression in youth and adolescents. The MFQ has been shown to have high criterion validity (Rhew et al., 2010), test–retest reliability (ICC = 0.76) (Wood et al., 1995), and excellent internal consistency (*α* = .91 to .93) (Thabrew et al., 2018).

#### 2.2.5 | The Children’s Depression Inventory (CDI)

CDI (Kovacs, 1992) is also a self-report questionnaire that assesses various symptoms of depression in youth and adolescents. The CDI has also been shown to have high test–retest reliability (0.81) and internal consistency (*α* = .85) (Figueras Masip et al., 2010; Ivarsson et al., 2006).

#### 2.2.6 | CONNERS

The Conners 3 ADHD INDEX-Parent (Conners, 2008) is a 10-item parent report scale that assesses ADHD symptoms. The Conners has shown high reliability (*α* = .92) and internal validity (KMO index value = 0.88) (Morales-Hidalgo et al., 2017).

### 2.3 | Image preprocessing

The “recon-all” pipeline from the FreeSurfer toolbox (Version 6.0; https://surfer.nmr.mgh.harvard.edu) was used to process the anatomical brain images (Dale et al., 1999; Fischl et al., 1999) and for estimating CV/subcortical volume measures. The version 6.0 of FreeSurfer toolbox implements an improved analytical method to estimate CV (Winkler et al., 2018). Processing of structural images involved basic image preprocessing steps, including head motion-correction, brain extraction, automated transformation to the standard MNI template space, volumetric segmentation into cortical and subcortical matter, intensity correction, and parcellation of the cerebral cortex into gyral and sulcal matter (Desikan et al., 2006). The technical details of preprocessing steps are documented in previous publications (Dale et al., 1999; Fischl, 2004; Fischl et al., 1999). To inspect the preprocessing accuracy, standard quality control steps were performed. These steps involved a careful visual inspection of raw structural images, skull-stripped brain volumes, and pial surfaces.

### 2.4 | Data analysis

#### 2.4.1 | Demographics characteristics and covariates

Group differences in sex were examined via *χ*^2^ test while those for age, IQ, intracranial volume (ICV), and GAD were examined via two samples *t* tests. Group differences in sex, age, and IQ obtained meant that the variable would be treated as a covariate in subsequent analyses. Because CV scales with head size, that is, ICV (Barnes et al., 2010), therefore, ICV was always included as a covariate in our analysis.

#### 2.4.2 | Network-wise group differences in CV

The Yeo’s Atlas (Yeo et al., 2011) was used to parcellate the whole brain into seven different networks (N1: Visual Network; N2: Somatomotor Network; N3: Dorsal Attention Network; N4: Ventral Attention Network; N5: Limbic Network, LN; N6: Frontoparietal Network, FPN; and N7: Default-Mode Network, DMN). The seven-network Yeo’s atlas used in the current study was previously generated using a stable clustering algorithm that was implemented on resting-state fMRI data from 1000 healthy control participants (Yeo et al., 2011). In other words, the seven networks of this parcellation are spatially distributed, that is, the location of two voxels within the same network need not be part of the same region.

Originally this parcellation was created using adult participants; however, other work has shown that the 400 regional parcellation that was based on the 7-network parcellation, used in the current study, reflects network organization in youth as well (J. Chen et al., 2020; Schaefer et al., 2018). Subject-wise and hemispheric-wise CV was evaluated for each network. CV data were averaged over both hemispheres for each identified network/region. Given the current hypotheses and focus of this study, only three networks (i.e., LN, FPN, and DMN) were included in further analysis. For the between group/main effect analysis and to identify the networks of interest among these three networks, multivariate analysis of covariance (MANCOVA; with sex, age, IQ, and ICV as potential covariates) was used to compare the network-wise hemispheric mean CV between the GAD and TD groups. A threshold of *p* ≤ .05 was used to interpret the network-wise CV differences between the GAD and TD groups. To interpret the group differences, we did not use multiple comparison correction across networks, as it is commonly agreed that it is important to understand what components (networks) are contributing to a significant group effect from MANCOVA. Therefore, we conducted a nonparametric *permutation test* in MATLAB R2021a (Krol, 2021; MATLAB 9.10 R2021a, 2021). The permutation test generates the distribution of test statistics under the null hypothesis and does not require any prior knowledge about that distribution. A total of 10,000 permutations (at critical *p* < .05) were used to obtain the null distribution.

#### 2.4.3 | Region-specific group differences in CV/subcortical volume

The anatomical locations and CV of regions within each identified network were extracted using *aparc.annot* (Desikan-Killany Atlas) (Desikan et al., 2006) and *mri_segstats* pipelines in FreeSurfer. Hemispheric mean subcortical volume was estimated from six subcortical areas, including the thalamus, caudate, putamen, pallidum, amygdala, and hippocampus, generated by FreeSurfer automated subcortical segmentation pipeline. However, only two subcortical structures, the amgydala and hippocampus were included in further analysis. The hippocampus was chosen because reduced hippocampal volume in adolescents or adults with GAD is one of the few consistent findings in the sMRI literature on GAD (Gold et al., 2017; Hettema et al., 2012; Moon et al., 2014). The amygdala was chosen because it is one of the regions most consistently referred to with respect to anxiety disorders generally as well as GAD (De Bellis et al., 2002; Etkin & Wager, 2007; Etkin et al., 2010; Gold et al., 2017; Strawn et al., 2015). Hemispheric mean CV of identified regions and volume of two subcortical regions (i.e., amygdala and hippocampus) were compared between the GAD and TD groups using MANCOVA with the same potential covariates (i.e., sex, age, IQ, and ICV). A threshold of *p* ≤ .05 was used to interpret the group-differences in region-specific CV/subcortical volume. To interpret the group differences, we did not use multiple comparison correction across regions, because again as it is important to understand what components (regions) are contributing to a significant group effect from MANCOVA.

### 2.5 | Follow-up analyses

#### 2.5.1 | Potential confounds: Impact of other major psychopathologies and prescribed medications

A number of our participants with GAD were co-morbid for major depressive disorder (MDD; *ßN* = 24), a common disorder comorbid with GAD (Remes et al., 2018; Shen et al., 2018) and attention deficit disorder (ADHD; *ßN* = 55) (Souza et al., 2005). In addition, a number of our patients with GAD were on medications during the time of the study. Given these potential confounds, a between-group MANCOVA—not only with ICV, but also with the inclusion of MDD diagnosis, ADHD Conners scores, and three prescribed medications (i.e., antipsychotic, SSRIs, and stimulants; scored 1 for “yes” or 0 for “no”) was conducted between the GAD and TD groups. Two additional MANCOVAs were run to identify potential group differences between individuals (a) with and without MDD and (b) with and without ADHD, both with the inclusion of ICV as a covariate.

We have also included two supplemental tables including the various groupings of comorbid disorders with the 81 adolescents with GAD.

## 3 | RESULTS

### 3.1 | Demographics characteristics and covariates

There were no significant group differences in sex (*χ*^2^ = 2.59, *p* = .11), age: *t* (191) = −1.40, *p* = .16, IQ: *t* (191) = 1.20, *p* = .23, or ICV: *t* (191) = 0.67, *p* = .50. Unsurprisingly, adolescents with GAD scored significantly higher than TD adolescents on SCARED GAD subscale scores; *t* (191) = −9.81, *p* < .001 (see Table 1).

### 3.2 | Network-wise group differences in CV

**FIGURE 1:**
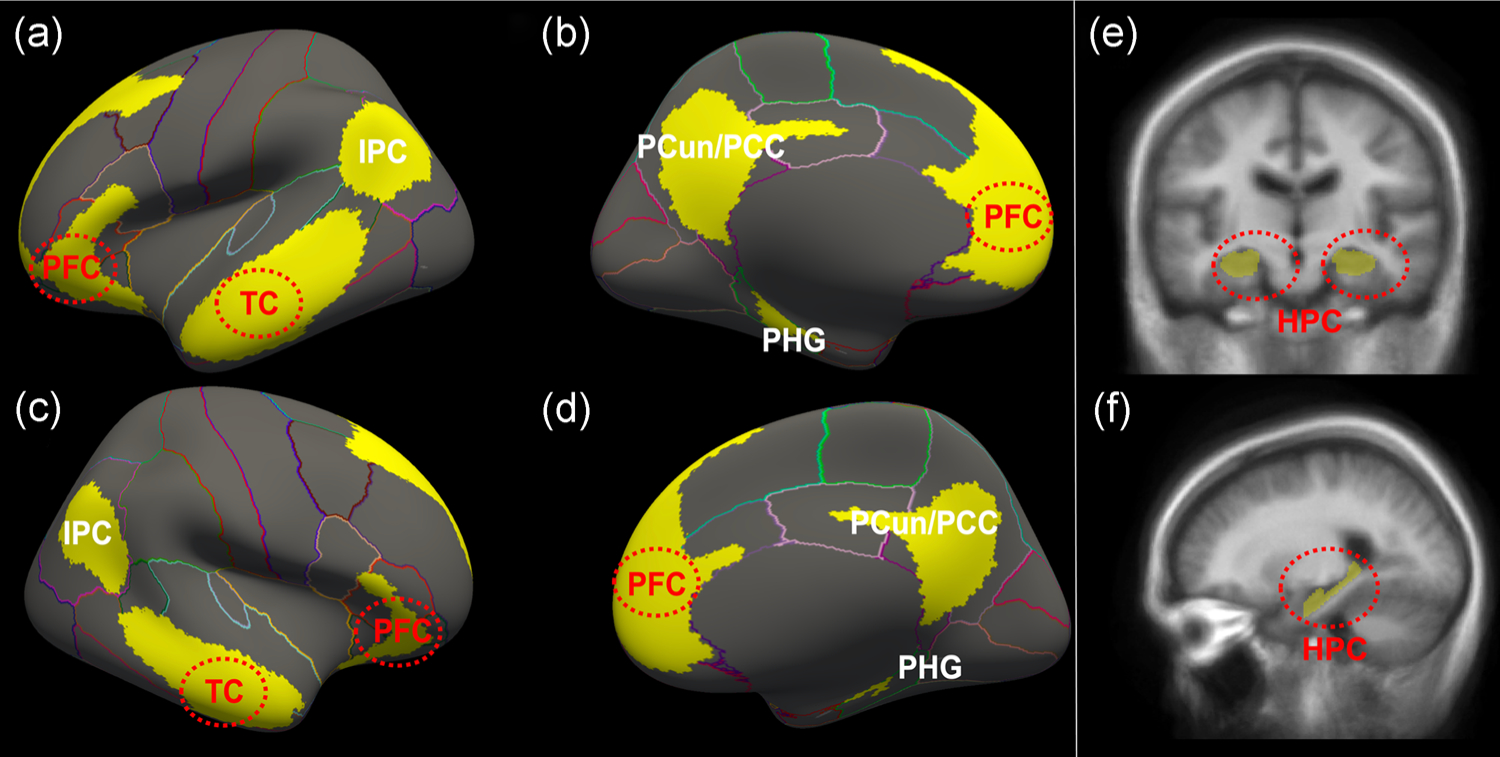
Identified cortical network and its component regions, and identified subcortical region. CV of the default-mode network (DMN) (left: a, b and right: c, d) were significantly different between the GAD sample and TD adolescents (GAD < TD). The DMN mainly constituted the inferior parietal cortex (IPC), temporal cortex (TC), prefrontal cortex (PFC), precuneus/posterior cingulate cortex (PCun/PCC), and parahippocampal gyrus (PHG). In (e, f), we show identified subcortical region (i.e., hippocampus, HPC) that showed significantly different subcortical volume between the GAD sample and TD adolescents (GAD < TD). Regions in red and dotted red circles represent specific regional significant findings within the DMN and subcortical region.

Our MANCOVA showed significant group differences in CV [*F* (3, 188) = 3.10, *p* = .03; *p*η^2^ = 0.05]. At *p* ≤ .05, there were significant group differences in hemispheric mean CV for the DMN [*F* (1, 193) = 7.96, *p* = .005, $\eta_{p}^{2}$ = 0.04]; the GAD group showed significantly lower CV than the TD group. None of the other networks showed significant group differences in CV at *p* ≤ .05 (see Table 2). Our *permutation test* rejected the null hypothesis and showed significant group differences only for the DMN CV (*p* = .02), and not for the LN (*p* = .20) or FPN (*p* = .10). The regions within the DMN are summarized in Table 3 and are shown in Figure 1.

Our MANCOVA showed significant group differences in CV [*F* (3, 186) = 2.70, *p* = .047; $\eta_{p}^{2}$ = 0.04] even after including age and sex as additional covariates (in addition to ICV). Again, there were significant group differences in hemispheric mean CV for the DMN [*F* (1, 193) = 5.61, *p* = .02, $\eta_{p}^{2}$ = 0.03]; the GAD group showed significantly lower CV than the TD group. None of the other networks showed significant group differences in CV at *p* ≤ .05.

### 3.3 | Region-specific group differences in CV/subcortical volume

Our MANCOVA showed significant group differences in CV/subcortical volume [*F* (7, 184) = 2.19, *p* = .04; $\eta_{p}^{2}$ = 0.08]. At *p* ≤ .05, there were significant group differences in hemispheric mean CV for the prefrontal cortex [*F* (1, 193) = 9.38, *p* < .005, $\eta_{p}^{2}$ = 0.05] and temporal cortex [*F* (1, 193) = 5.06, *p* = .03, $\eta_{p}^{2}$ = 0.03], and subcortical volume of the hippocampus [*F* (1, 193) = 4.74, *p* = .03, $\eta_{p}^{2}$ = 0.02] (see Figure 1); the GAD group showed significantly lower CV/subcortical volume than the TD group. None of the other regions (other than PFC, TC, and HPC) showed significant group differences at *p* ≤ .05 (see Table 4).

### 3.4 | Follow-up findings

Our follow-up MANCOVA mirrored the main analysis, showing significant group differences in hemispheric mean CV of DMN [*F* (1, 148) = 4.853, *p* = .029, $\eta_{p}^{2}$ = 0.034] with the inclusion of MDD, ADHD scores, ICV, and three prescribed medications (i.e., antipsychotic, SSRIs, and stimulants; as covariates). None of the other networks showed significant group differences in CV. Our two additional follow-up MANCOVAs showed no group differences in hemispheric mean CV of DMN for those with MDD vs. those without MDD [*F* (1, 78) = 0.60, *p* = .44, $\eta_{p}^{2}$ = 0.01] and those with ADHD vs. those without ADHD [*F* (1, 78) = 0.54, *p* = .46, $\eta_{p}^{2}$ = 0.01]. This further confirms that there was no significant contribution of both MDD and ADHD diagnoses to our main results.

#### 3.4.1 | Group differences in CT and CSA

Our MANCOVA showed nonsignificant group differences in CT [*F* (3,189) = 1.16, *p* = .32; $\eta_{p}^{2}$ = 0.02] and CSA [*F* (3, 188) = 1.30, p = .28; $\eta_{p}^{2}$ = 0.02].

## 4 | DISCUSSION

The goal of this study was to examine CV/subcortical volume in individuals with GAD and TD adolescents. Adolescents with GAD were found to have significantly lower CV for the DMN, as well as the prefrontal and temporal regions of the DMN, compared with TD adolescents. There were no other significant group differences in the two other networks (i.e., LN and FPN). With respect to subcortical regions, individuals with GAD showed significantly lower subcortical volume of the hippocampus relative to TD adolescents. No significant group differences in hemispheric mean subcortical volume for the amygdala were observed.

Our investigation of group differences in CV within the cortical networks was somewhat exploratory—even if networks likely to show structural abnormalities could be hypothesized. Not only has the previous literature reported relatively inconsistent findings with respect to group differences in CV but it has also not typically taken a network-based approach to analysis. Our results indicated that GAD in adolescents is associated with significantly lower CV for the DMN and its prefrontal and temporal regions. Previous studies have reported atypical structural findings within regions comprising the DMN though the directionality of these findings has been inconsistent (Y. Chen et al., 2020; Gold et al., 2017; Molent et al., 2018; Schienle et al., 2011; Strawn et al., 2013). Specifically, studies have reported increased (Gold et al., 2017) and decreased vmPFC volumes (Y. Chen et al., 2020), increased (Schienle et al., 2011) and decreased dmPFC volumes (Y. Chen et al., 2020; Molent et al., 2018), decreased PCC volumes (Strawn et al., 2013), and increased (De Bellis et al., 2002; Strawn et al., 2013) and decreased (Y. Chen et al., 2020; Moon et al., 2014, 2015) temporal cortex volumes. It is unclear why there are inconsistent findings as compared with some previous DMN-relevant sMRI findings of patients with GAD. However, it is worth noting that the studies by Schniele et al. and DeBellis et al. had relatively small sample sizes (16 and 13 patients with GAD, respectively) and the Gold et al. findings came from a mixed sample of adolescents with anxiety disorders (only 73% presented with GAD).

A number of studies have examined task-related functional connectivity in patients with GAD and reported atypical integrated signaling between the amygdala, PFC, and anterior cingulate cortex (ACC) (Etkin et al., 2010; Monk et al., 2008). More recently, whole-brain resting-state analysis studies have begun to consistently identify atypical integrated signaling within the DMN in patients with GAD (Y. Chen et al., 2020; Rabany et al., 2017; Xiong et al., 2020; Yao et al., 2017). As such, the current findings of structural abnormalities within the neural systems making up the DMN are consistent with these previous resting-state studies. The DMN has been implicated in a number of functions that have clear relevance to GAD; specifically, emotion regulation and self-referential processing (Greicius et al., 2003; Gusnard et al., 2001). Disruption in either process might be associated with symptoms seen in GAD with worry perhaps particularly relating to disrupted self-referential processing (Makovac et al., 2016).

Stress has been shown to have a negative impact on the prefrontal cortex (A. F. T. Arnsten, 2009). Even mild stress can weaken the prefrontal cortex function, including decision making and emotion regulation (A. Arnsten et al., 2012). Specifically, the ventral medial prefrontal cortex has multiple connections to subcortical regions that are involved in emotion regulation (Motzkin et al., 2015). The temporal pole has also been shown to be associated with various forms of emotional regulation (Olson et al., 2007). Volume differences in these regions could suggest an inability to effectively regulate emotions and make decisions, both shown to be affected in individuals with GAD.

In line with our prediction, the current study also observed that patients with GAD showed reduced hippocampal volumes relative to TD adolescents. These findings replicate previous results reporting reduced hippocampal volumes in adolescents and adults with GAD (Gold et al., 2017; Hettema et al., 2012; Moon et al., 2014). Some previous work has not reported hippocampal structural abnormalities in patients with GAD (De Bellis, 2000; De Bellis et al., 2002; Milham et al., 2005)—but none of these studies reported increased hippocampal volumes (unlike findings for other regions where increased and decreased volumes have been reported). As such, the current findings implicating structural perturbations in the hippocampus, in the context of previous similar findings (Gold et al., 2017; Hettema et al., 2012; Moon et al., 2014), suggest that increased attention should be paid to functional perturbations of this region. The hippocampus has been associated with emotion and fear processing, and frequently seen in the neurocircuitry of anxiety disorders (Duval et al., 2015). It has also been shown that chronic stress has detrimental effects on hippocampal structure (Conrad et al., 2001). Stress resulting from constant anxiety could cause a reduction in hippocampal volume, leading to abnormal emotion regulation and processing, which could then result in exacerbated anxiety symptoms.

Amygdala volumes in individuals with GAD have been inconsistent, such that studies have shown an increase (De Bellis, 2000; Etkin & Wager, 2007; Schienle et al., 2011), decrease (Milham et al., 2005; Strawn et al., 2013), and no change in volume (Milham et al., 2005). In the present study we did not see any significant differences in the amygdala volume.

Four caveats in this study should be noted. First, GAD is highly comorbid with multiple diagnoses including MDD (Remes et al., 2018; Shen et al., 2018), and ADHD (Souza et al., 2005), which was also seen in the current sample. As such, it could be argued that the comorbid MDD or ADHD diagnoses were contributing to our results. However, our two follow-up network-focused MANCOVAs with MDD and ADHD scores as additional covariates mirrored the results of the primary results. Our follow-up analyses also showed that the MDD and ADHD diagnoses had no significant impact on our findings. Second, a portion of our participants with GAD were on medications during the time of the study. We ran a follow-up analysis with psychiatric medications as additional covariates in our MANCOVA and results were proximal to the main analysis. However, there is considerable heterogeneity in specific medication, dose, length of time on medication within the adolescents with GAD. This is difficult to capture statistically. But the commonality within the group of adolescents with GAD is their GAD diagnosis. As such, we would argue that it is more plausible that our results relate to the diagnosis rather than a heterogeneous range of treatments. It should also be noted that the adolescents received residential care treatment at BTNRH; however, they were scanned close to their arrival, and it is unlikely that this treatment contributed to any structural differences. Third, while the TD adolescents did not demonstrate psychiatric diagnoses, the psychiatric status of their first-degree relatives was not ascertained. Note though our approach decreased the likelihood of observing significant group differences as there may be more “noise” in the TD data (corresponding to abnormalities associated with the psychiatric status of first-degree relatives). Indeed, the current approach should identify structural abnormalities relevant to understanding GAD—it avoids identifying structural abnormalities that are not associated with GAD per se (as they did not engender GAD in the HCs) but are associated with psychiatric psychopathology in first-degree relatives. Fourth, it should be noted that our network-based group differences were significant for CV but not for CT and CSA. Prior work mitigates this concern and indicates the joint analysis of CT and CSA in terms of CV increases the power to simultaneously quantify the effects of CT and CSA (Rimol et al., 2012). In accordance with that the current data indicate neither CT nor CSA contributes significantly, but the joint analysis of CT and CSA in terms of CV shows significant network-based group differences.

In conclusion, our study revealed a decrease in CV within the DMN, and also within the prefrontal cortex, temporal cortex, and hippocampus. These findings can provide an insight to structural neural deficits in adolescents with GAD and provide an understanding of better treatment methodologies.

## Supplementary Material

tS2

tS1

## Figures and Tables

**TABLE 1:** Demographics characteristics and group differences

Characteristics	GAD group (*N* = 81)	TD group (*N* = 112)	Statistics
Sex	42F/39M	45F/67M	*χ*^2^ = 2.59, *p* = .11
Age	15.98 (SD = 1.65)	15.63 (SD = 1.74)	*t* (191) = −1.40, *p* = .16
IQ	101.90 (SD = 12.74)	103.94 (SD = 10.92)	*t* (191) = 1.20, *p* = .23
ICV (×10^6^) in mm^3^	1.50 (SD = 0.15)	1.51 (SD = 0.16)	*t* (191) = 0.67, *p* = .50
Psychopathologies			
MDD	24 (29.6%)	0	–
SAD	44 (54.3%)	0	–
PTSD	30 (37%)	0	–
CD	40 (49.4%)	1 (.9%)	–
ADHD	55 (67.9%)	2 (1.8%)	–
Medications			
Antipsychotic	6 (7.4%)	2 (1.8%)	–
SSRIs	25 (30.9%)	4 (3.6%)	–
Stimulants	12 (14.8%)	3 (2.7%)	–
Assessments			
GAD score	9.68 (SD = 4.95)	4.03 (SD = 3.01).	*t* (191) = −9.81, *p* < .001
MFQ	20.10 (SD = 14.35)	4.66 (SD = 4.60)	*t* (156) = −8.76, *p* <.001
CDI	15.46 (SD = 9.26)	4.16 (SD = 3.88)	*t* (86.50) = −9.52, *p* <.001
Connors	5.48 (SD = 6.33).	0.7143 (SD = 2.57)	*t* (99.21) = −6.41, *p* <.001

Abbreviations: ADHD, attention deficit hyperactivity disorder; CD, conduct disorder; CDI, Children's Depression Inventory; GAD, generalized anxiety disorder; ICV, intercranial cortical volume; IQ, intelligent quotient; MDD, major depressive disorder; MFQ, Mood and Feelings Questionnaire; PTSD, post-traumatic stress disorder; SAD, social anxiety disorder; SD, standard deviation; SSRIs, selective serotonin reuptake inhibitors; TD, typically developing.

a GAD subscore on the Screen for Child Anxiety Related Disorders (SCARED) scale (Birmaher et al., 1997199)

**TABLE 2:** Multivariate analysis of covariance (MANCOVA): Differences in network-specific CV

	Hemispheric mean CV		
Networks	*F* (1,193)	*p*	$\eta_{p}^{2}$
Limbic network (LN)	1.24	.27	0.01
Frontoparietal network (FPN)	3.13	.08	0.02
Default-mode network (DMN)	7.96	.005*	0.04

Abbreviations: CV, cortical volume; GAD, generalized anxiety disorder; TD, typically developing.

* *p* ≤ .05 (uncorrected) (GAD < TD).

**TABLE 3:** Anatomical regions within the DMN

Networks	Regions
Left DMN	Parietal cortex (ParC), temporal cortex (TC), prefrontal cortex (PFC), precuneus/posterior cingulate cortex (PCun/PCC), parahippocampal gyrus (PHG)
Right DMN	Parietal cortex (ParC), temporal cortex (TC), ventral prefrontal cortex (vPFC), medial prefrontal cortex (mPFC), precuneus/posterior cingulate cortex (PCun/PCC), parahippocampal gyrus (PHG)

Abbreviation: DMN, Default Mode Network.

**TABLE 4:** Multivariate analysis of covariance (MANCOVA): Differences in region-specific CV within the DMN and subcortical volume

	CV/sub-cortical volume		
Regions	*F* (1,193)	*p*	$\eta_{p}^{2}$
Prefrontal cortex (PFC)	9.38	.003*	0.05
Temporal cortex (TC)	5.06	.03*	0.03
Precuneus/posterior cingulate cortex (PCun/PCC)	0.56	.46	0.00
Parietal cortex (ParC)	2.15	.14	0.01
Parahippocampal gyrus (PHG)	0.01	.93	0.00
Amygdala	1.57	.21	0.01
Hippocampus	4.74	.03*	0.02

Abbreviations: CV, cortical volume; DMN, Default Mode Network; GAD, generalized anxiety disorder; TD, typically developing.

* *p* ≤ .05 (GAD < TD).

## Data Availability

The data that support the findings of this study are available from the corresponding author upon reasonable request. The data are not publicly available due to IRB restrictions.
