# Supplementary material for: Reduced cortical volume of the default mode network in adolescents with generalized anxiety disorder
Source: Depress Anxiety. Author manuscript; Available in PMC 2023 Jun 1. (PMC9246827; doi:10.1002/da.23252)
Supplement: tS2 [file NIHMS1788816-supplement-tS2.docx]

| **Supplemental Table 2: GAD SCARED Scores and Comorbidities (Groupings)** | | |
| --- | --- | --- |
| **Psychopathologies** | **With (N)** | **SCARED Score** |
|  |  |  |
| **GAD only** | 6 | 8.5 (SD=5.89) |
| **MDD** | 0 | -- |
| **SAD** | 5 | 11.2 (SD=4.82) |
| **PTSD** | 2 | 9 (SD=1.41) |
| **CD** | 0 | -- |
| **ADHD** | 8 | 5 (SD=5.0) |
| **MDD + SAD** | 3 | 14.67 (SD=2.52) |
| **MDD + PTSD** | 1 | 2 |
| **MDD + ADHD** | 2 | 12 (SD=7.07) |
| **SAD + PTSD** | 1 | 12 |
| **SAD + CD** | 3 | 6 (SD=4.58) |
| **SAD + ADHD** | 5 | 7.6 (SD=3.36) |
| **PTSD + CD** | 2 | 8 (SD=4.24) |
| **CD + ADHD** | 8 | 7.5 (SD=4.07) |
| **MDD + SAD + PTSD** | 1 | 2 |
| **MDD + SAD + ADHD** | 1 | 16 |
| **MDD + PTSD + CD** | 1 | 1 |
| **MDD + PTSD + ADHD** | 2 | 14.5 (SD=2.12) |
| **SAD + PTSD + CD** | 1 | 10 |
| **SAD + PTSD + ADHD** | 2 | 9 (SD=2.83) |
| **SAD + CD + ADHD** | 5 | 7.8 (SD=1.1) |
| **PTSD + CD + ADHD** | 4 | 7.25 (SD=3.77) |
| **MDD + SAD + PTSD + ADHD** | 2 | 16 (SD=2.83) |
| **MDD + SAD + CD + ADHD** | 5 | 13.6 (SD=2.07) |
| **MDD + PTSD + CD + ADHD** | 1 | 13 |
| **SAD + PTSD + CD + ADHD** | 5 | 14.6 (SD=3.91) |
| **MDD + SAD + PTSD + CD + ADHD** | 5 | 15 (SD=4.24) |

Key to table. GAD=Generalized Anxiety Disorder; W/O= without; MDD= Major Depressive Disorder; SAD=Social Anxiety Disorder; PTSD= Post Traumatic Stress Disorder; CD=Conduct Disorder; ADHD=Attention Deficit Hyperactivity Disorder; SD= Standard Deviation

^GAD sub-score on the Screen for Child Anxiety Related Disorders (SCARED) scale
