# Supplementary material for: Reduced cortical volume of the default mode network in adolescents with generalized anxiety disorder
Source: Depress Anxiety. Author manuscript; Available in PMC 2023 Jun 1. (PMC9246827; doi:10.1002/da.23252)
Supplement: tS1 [file NIHMS1788816-supplement-tS1.docx]

**Supplemental Table 1: GAD SCARED Scores and Comorbidities with GAD**

| **GAD Group (N=81)** | | | | | | |
| --- | --- | --- | --- | --- | --- | --- |
| **Psychopathologies** | **With (N)** | **SCARED Score** | **Without (N)** | **SCARED Score** |  | **Statistics** |
|  |  |  |  |  |  |  |
| **MDD** | 24 | 12.75 (SD=5.16) | 57 | 8.39 (SD=4.28) |  | *t* (79) = -3.94 |
|  |  |  |  |  |  | p<0.001 |
| **SAD** | 44 | 9.62 (SD=5.35) | 37 | 9.62 (SD=5.35) |  | *t* (79) = -.095 |
|  |  |  |  |  |  | p=.925 |
| **PTSD** | 30 | 9.57 (SD=5.71) | 51 | 9.75 (SD=4.50) |  | *t* (79) = 156 |
|  |  |  |  |  |  | p=.877 |
| **CD** | 40 | 9.60 (SD=5.05) | 41 | 9.76 (SD=4.91) |  | *t* (79) = 141 |
|  |  |  |  |  |  | p=.888 |
| **ADHD** | 55 | 9.58 (SD=5.13) | 26 | 9.89 (SD=4.65) |  | *t* (79) = .255 |
|  |  |  |  |  |  | p=.799 |

Key to table. GAD=Generalized Anxiety Disorder; W/O= without; MDD= Major Depressive Disorder; SAD=Social Anxiety Disorder; PTSD= Post Traumatic Stress Disorder; CD=Conduct Disorder; ADHD=Attention Deficit Hyperactivity Disorder; SD= Standard Deviation

^GAD sub-score on the Screen for Child Anxiety Related Disorders (SCARED) scale
